# Silica Hazards in Engineered Stone Countertop Production: Worker Experiences and Challenges in Los Angeles

**DOI:** 10.1002/ajim.70010

**Published:** 2025-07-28

**Authors:** Jane C. Fazio, Sandra R. Garcia, Ivy R. Torres, Sheiphali A. Gandhi, Kristin J. Cummings, Russell G. Buhr, Kevin Riley, Ninez Ponce, Arturo Vargas Bustamante, Beth Glenn

**Affiliations:** 1 Department of Health Policy and Management, Fielding School of Public Health, University of California, Los Angeles, Los Angeles, California, USA; 2 Division of Pulmonary, Critical Care, and Sleep Medicine, Department of Medicine, David Geffen School of Medicine, University of California, Los Angeles, Los Angeles, California, USA; 3 Department of Community Health Sciences, Fielding School of Public Health, University of California, Los Angeles, Los Angeles, California, USA; 4 Labor Occupational Safety and Health Program, University of California, Los Angeles, Los Angeles, California, USA; 5 Division of Pulmonary, Critical Care, Allergy and Sleep Medicine, Department of Medicine, University of California, San Francisco, San Francisco, California, USA; 6 Division of Occupational, Environmental and Climate Medicine, University of California, San Francisco, San Francisco, California, USA; 7 Occupational Health Branch, California Department of Public Health, Richmond, California, USA; 8 Center for the Study of Healthcare Innovation, Implementation, and Policy, Health Systems Research, Greater Los Angeles Veterans Affairs Healthcare System, Los Angeles, California, USA; 9 Latino Politics and Policy Institute, University of California, Los Angeles, Los Angeles, California, USA; 10 UCLA Kaiser Permanente Center for Health Equity, University of California, Los Angeles, Los Angeles, California, USA; 11 Jonsson Comprehensive Cancer Center, University of California, Los Angeles, Los Angeles, California, USA

**Keywords:** engineered stone, fabrication workshops, hazardous work, personal protective equipment, public health, respirable crystalline silica, silicosis

## Abstract

**Background:**

Silicosis is a progressive occupational lung disease caused by inhalation of respirable crystalline silica. Stone countertop workers are at risk of developing silicosis associated with engineered stone (ES) due to its high silica content and widespread use. This qualitative study assessed countertop workers' knowledge, attitudes, and practices regarding silica hazards.

**Methods:**

We recruited 20 participants for semi-structured interviews through purposive sampling of workplaces in Los Angeles, California. Eligibility requirements: employment in countertop fabrication, exposure to engineered stone, ≥ 18 years old, and English or Spanish proficiency. Interviews were recorded, translated, and transcribed. Two researchers employed thematic and content analysis to code transcripts using both inductive and deductive methods.

**Results:**

All respondents were Latino men, 90% of whom were foreign-born, with a median age of 44 years and a median work tenure of 20 years. We identified five themes: (1) *Hazardous work environments:* Respondents regularly engaged in dry-cutting practices (68%), and half reported that their employers did not reliably provide respirators. (2) *Insufficient training and educational resources:* Respondents possessed basic knowledge that dust caused lung disease (90%); however, most acquired knowledge through word-of-mouth or media. (3) *Employment vulnerability:* Workers feared being fired for raising safety concerns. (4) *Workforce entrapment:* Although many workers considered leaving the industry, they were hindered by a lack of legal immigration status or alternative skills, as well as the presumed lower pay in a new industry. (5) *Worker-identified solutions:* Workers suggested wet cutting, workplace training, and regulation of ES, including its elimination.

**Conclusion:**

The results support continued policy, regulatory, and enforcement efforts aimed at minimizing silica exposure. Workers recommended workplace and upstream solutions, such as eliminating ES.

## 1 | Introduction

Silicosis is a progressive and incurable occupational lung disease caused by inhalation of aerosolized silica particles. Silicosis can lead to severe disability and death, and at the moment, there are limited treatment options, with the most effective being lung transplantation. Silicosis is typically associated with occupations such as mining and sandblasting; however, in the last decade, physicians have documented a surge in silicosis among workers who cut, grind, and fabricate engineered stone countertops [1–7]. Since 2019, California public health officials have identified over 321 cases of silicosis associated with the fabrication of engineered stone countertops, with cases rising exponentially [6, 8–10]. Disease occurrence has risen in tandem with an increase in consumer demand for engineered stone countertops, which have at least twice or greater the silica content of natural stones like marble or granite, posing increased risk to workers [11, 12]. These engineered stones are made of crushed quartz bound together by an adhesive resin and have dominated the US consumer market over the last decade due to their durability, affordability, and ease of maintenance compared to natural stone [13, 14].

Nearly all affected stone countertop workers in the United States and California reported to date are Latino immigrants, often undocumented, from Mexico or Central America [1, 6, 15]. They frequently work under precarious employment arrangements characterized by low wages, low levels of workplace agency, and limited legal protections and social benefits [6, 16]. Consequently, they may be reluctant to demand improved working conditions due to fear of dismissal or reprisal [15]. A Latino immigrant workforce is also more likely to be uninsured or underinsured, meaning that they tend to delay or altogether forego medical care, more often presenting with advanced silicosis due to medical care being cost-prohibitive, medical systems being difficult to navigate, or lack of English proficiency among a group whose primary language is Spanish [1, 6, 7, 17]. In a case-series analysis documenting the first cases of silicosis from engineered stone in California, nearly half of the countertop workers diagnosed with silicosis were identified during emergency department visits, rather than through primary care visits or a medical surveillance mechanism [6].

Globally, nearly all research on the health effects of engineered stone fabrication has focused on quantifying the number of workers diagnosed with silicosis or measuring the average level of exposure to respirable crystalline silica on an average day through air sampling [3, 18]. Several of the existing studies were published outside of the United States, notably in Australia, Spain, and Israel, as they identified silicosis cases among engineered stone fabricators earlier on [3, 18]. These studies also have a primary focus on the immediate health impacts instead of capturing workers’ perspectives on silicosis risks and challenges in addressing risks [3, 18]. Prior research exploring in-depth perspectives of engineered stone countertop fabrication workers is limited to one qualitative study in Spain; its findings are consistent with quantitative observations in the United States [19, 20]. There has been no research exploring the unique challenges faced by a largely Latino immigrant workforce in the United States. As the epicenter of the engineered stone fabrication industry in the United States, California offers a valuable opportunity to capture the cumulative disadvantages experienced by this marginalized workforce from the workers’ own perspective.

For these reasons, there is an urgent need to understand the unique experiences of the predominantly immigrant Latino workforce fabricating countertops in the United States, and the societal and interpersonal forces that may be contributing to the resurgence of silicosis in this population. Our goals were to use a qualitative approach guided by the socio-ecological framework to investigate stone countertop fabricators’ (1) working conditions, (2) knowledge about the hazards of silica dust inhalation and prevention strategies, (3) barriers to the implementation of safe practices such as wet cutting and respirator use in the workplace, and (4) the compounding disadvantages associated with immigration status among an already marginalized working population.

## 2 | Methods

### 2.1 | Study Design

We used individual in-depth semi-structured interviews guided by the socio-ecological framework to elicit workers’ perspectives and attitudes on their workplace silicosis hazards and prevention measures. The interview protocol was piloted with one patient who was not a participant in the study. The interview questions were further improved after administering the protocol to the first five study participants. The University of California, Los Angeles Institutional Review Board (IRB) approved this study (PRE#23-003209).

#### 2.1.1 | Theoretical Framework

**FIGURE 1 |:**
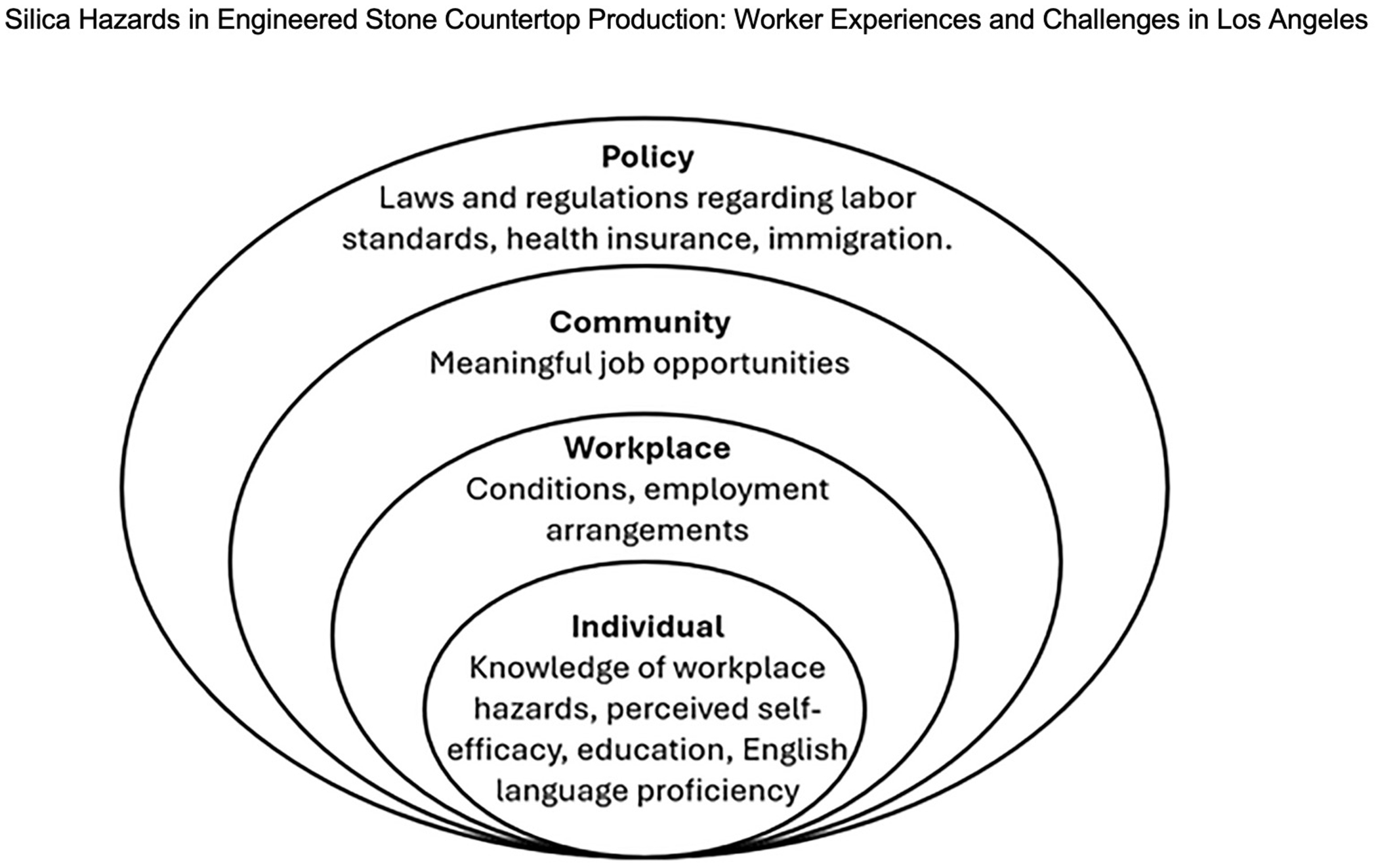
Socioecological framework for occupational health adapted from Bronfenbrenner’s original socioecological model.

We applied the socio-ecological model (Figure 1) as a guiding framework for this study [21, 22]. The socio-ecological model focuses on people’s interactions with factors in their physical and sociocultural environments that intersect across multiple levels of influence to develop effective health promotion interventions [22]. We employed interview questions within all four domains of influence—policy, community, workplace, and individual—to better understand the influences of each domain on the risk of over-exposure to silica dust in the workplace. The Policy level encompasses local, state, and national laws and policies that can bolster or limit health [22]. *Community* level factors are relationships among individuals, groups, and organizations and their accompanying social customs [22]. The *Workplace* includes institutional-level factors that help or hinder recommended health behaviors [22]. The *Individual* level is composed of intrapersonal level characteristics and skills that evolve through continued interactions with various social and environmental factors across a person’s developmental history [22].

#### 2.1.2 | Sample and Recruitment

We employed two non-probability sampling techniques often used in qualitative research to engage niche hard-to-reach subsets of the population and for studies with small samples—purposive and snowball sampling [23, 24]. Purposive sampling strategies were implemented by leveraging the ongoing silicosis prevention outreach efforts of the Los Angeles County Department of Public Health (LACDPH) and community partners to recruit respondents from workers in stone countertop fabrication [23]. Respondents were either recruited at worker centers or during site visits to fabrication shops throughout Los Angeles County. During these outreach efforts, interested workers were asked to share the study information with other workers in their network (snowball sampling) [24]. We screened interested participants for eligibility using the following criteria: current or prior employment as a stone countertop worker, regularly worked with engineered stone, 18 years or older, and fluent in Spanish or English.

Data collection occurred between August and December 2023. Interviews lasted between 30 and 45 min and each participant was compensated for their time. Investigators conducted interviews in person, at a park or community center, except for one interview that was completed by phone. Researchers accommodated workers’ schedules to enhance the representativeness of our sample. Before the start of the interview, researchers obtained verbal informed consent, and participants completed a demographic and occupational survey. Due to concerns for confidentiality, specific information regarding workers’ employers was not collected, including company name, company address, or OSHA citations/air sampling data.

The semi-structured interview guide explored topics including work environment, work practices related to silica dust, awareness of the hazards of engineered stone, personal experiences with silicosis and work-related illnesses or injuries, exposure reduction strategies, perceived influence in the workplace, and perceived ease of leaving the industry (Table 1). The interview guide was pilot-tested and revised early during the data collection to improve clarity. The domains assessed during interviews were informed by prior published work in industrial hygiene, occupational health and medicine, the 2016 Cal/OSHA Silica Standard, and the 2023 Emergency Temporary Standard (ETS) [6, 8, 19, 25, 26]. Interview guide can be found in the Supplementary Appendix I.

#### 2.1.3 | Data Analysis

Interviews were audio-recorded, and audio files were reviewed for completeness. A third-party professional service transcribed and translated audio files. Bilingual researchers reviewed transcripts for completion and accuracy. Transcripts were uploaded into Dedoose version 9.0.17 for coding and data analysis [27]. We employed both content and thematic analysis to examine major themes within each domain. Content analysis provided an objective method that allowed us to systematically categorize and quantify specific phenomena by making inferences from written data [28, 29]. Thematic analysis, one of the most commonly used qualitative analysis methods, provided a flexible but structured framework that allowed us to identify, analyze, and interpret themes within the interview data to provide a deeper understanding of participants’ experiences [30].

**FIGURE 2 |:**
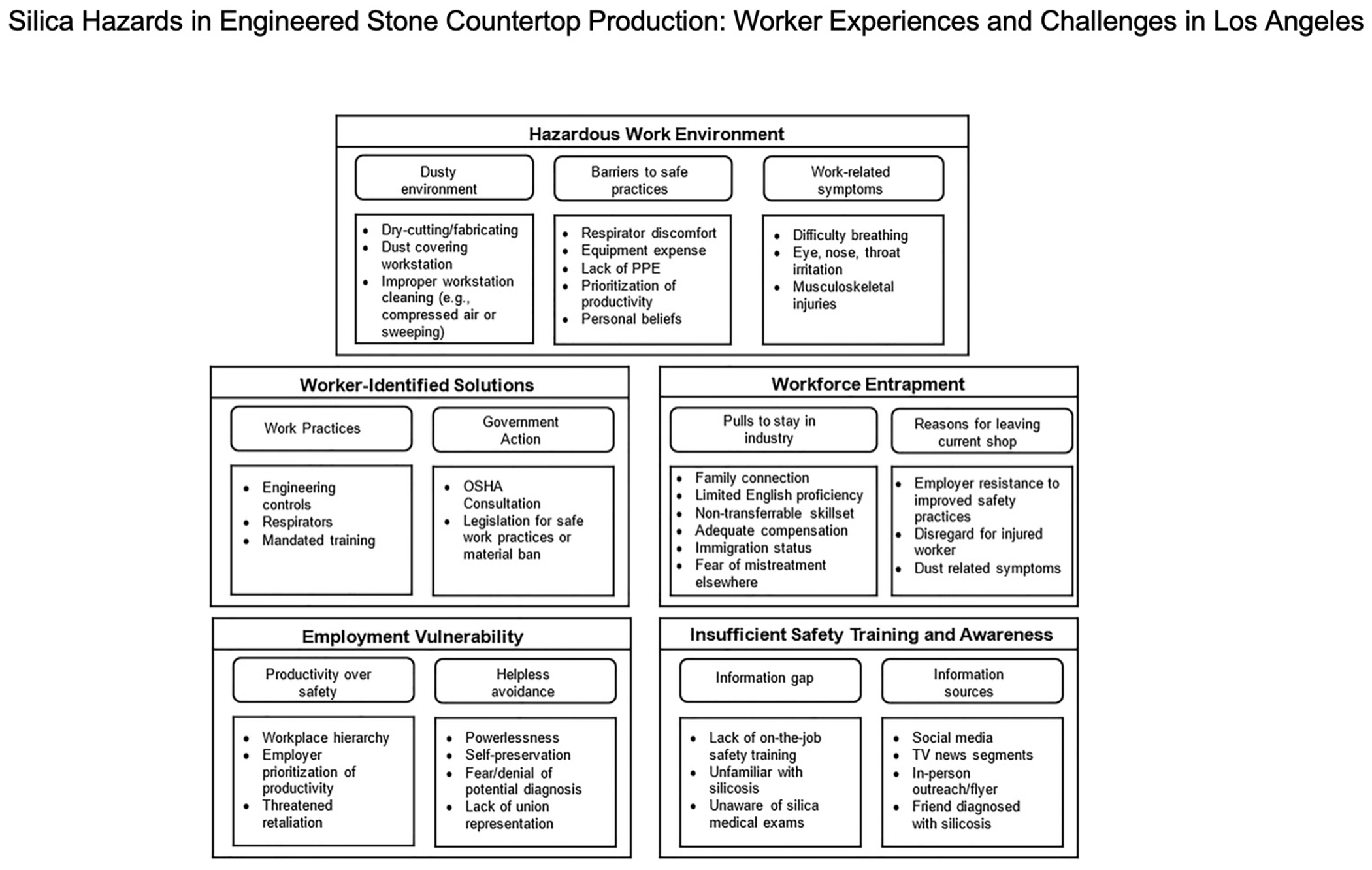
Structure of major themes including first and second order codes.

Content and thematic analysis were facilitated by a hybrid deductive and inductive coding system approach. The deductive reasoning approach included predetermined themes previously described in the literature such as high silica dust exposures due to inadequate protections and lack of knowledge and training about engineered stone [19, 20, 31, 32]. An inductive reasoning approach allowed us to draw further conclusions from the data that were informed by our socio-ecological model. The inductive approach guided our application of first-order codes which were then grouped into second-order codes based on commonality [31, 32]. An initial codebook was developed based on major domains explored in our guides (Figure 2). Two analysts (J.C.F. and S.G.) independently reviewed an initial subset of four transcripts which allowed for the reconciliation of differences in codes and reflexive changes in code definitions.

### 2.2 | Research Team and Reflexivity

Four bilingual research staff S.G. (MPH, PhD Student), I.T. (PhD) and X.Z. (MPH student), J.C.F. (Pulmonary Physician and Project Principal Investigator [P.I.]), conducted the interviews in Spanish or English, depending on the participant’s language preference. To minimize potential bias, all participants were recruited outside of the healthcare system to ensure that the PI, an expert in treating silicosis, had no prior relationship or familiarity with participants.

All researchers underwent training which included an overview of the study objectives, the interview guide, and instruction on conducting qualitative interviews. The training emphasized managing sensitive topics and ensuring consistency across interviews. Mock interviews were conducted to practice these skills, followed by feedback sessions to refine techniques. Additionally, interviewers were briefed on ethical considerations, including obtaining informed consent and maintaining confidentiality. The research team was introduced to the silicosis prevention outreach team to ensure the development of a good rapport and productive strategies for participant recruitment. Before the interviews, researchers informed participants about the growing number of workers in the stone fabrication industry developing lung disease. They explained that the purpose of the study was to gain a deeper understanding of work experiences and the factors contributing to these health outcomes.

## 3 | Results

Forty-five engineered stone countertop workers expressed interest and were screened for the study, of which only two were ineligible. Of those screened, 20 (44%) completed interviews, 19 in person and one by phone. Nineteen interviews were completed in Spanish and one in English.

### 3.1 | Participant Characteristics

All respondents identified as Latino males and had a median age of 44 years (IQR 35–49 years). Median work tenure in stone countertop production in the United States was 20 years (Table 2). Unsafe work practices were commonly reported; 68% (13/19) endorsed regular dry cutting, 50% (9/18) stated their employers did not reliably provide appropriate respirators, and only 5% (1/20) described having undergone any formal safety training. Half of the participants endorsed having a work-related injury or symptom. Eighty percent (8/10) of those who reported a work-related injury or symptom experienced respiratory symptoms and 20% (2/10) reported musculoskeletal injury/trauma. None reported having been previously diagnosed with silicosis. Seventy-five percent (15/20) of workers had a usual source of healthcare and 74% (14/19) had some form of health insurance. Ninety percent (18/20) knew that dust exposure from cutting engineered stone caused lung disease. However, 39% (7/18) had acquired that knowledge exclusively through a coworker by word of mouth. A third (33%, 6/ 18) had acquired that health knowledge in combination with media/social media, and less commonly through formal education by a public health or healthcare professional (28%, 5/18).

### 3.2 | Themes

The analysis revealed five major themes related to the study objectives: (1) hazardous work environments, (2) insufficient training and educational resources, (3) employment vulnerability, (4) workforce entrapment, and (5) worker-identified solutions, see Figure 2 for derivation of themes.

#### 3.2.1 | Hazardous Work Environments

Workers reported being regularly exposed to high concentrations of silica dust (60%, 12/20), a lack of effective dust removal/collection tools (67%, 12/18), and a lack of appropriate personal protective equipment (PPE) (60%, 12/20). Workers commonly identified other hazards associated with engineered stone fabrication such as exposures to chemical irritants (e.g., acetone), with associated respiratory and skin symptoms, as well as musculoskeletal injuries incurred on the job. Some (35%, 7/20) expressed fears of having silicosis or developing it in the future and feeling pressured to engage in work practices that prioritized expediency over worker safety.

Workers described that they and their fabrication shops were often covered in silica dust, which was thought to cause irritation and breathing difficulty (67%, 12/18). Reporting of exposure to silica dust was not verified through air sampling methods, but instead gathered from worker accounts of primarily working with engineered stone countertops, which were identified by brand. One stated “there is dust everywhere; you go to eat there is dust, you go to the bathroom there is dust, to the warehouse there is dust everywhere there is dust.” Another worker stated,

What happens with the dust is that you’re covered in it. Your face, your eyes, your nose. You’re covered in this dust. It gets in your hair too. And you can’t even touch your face because if you do, it goes in your nose. And that’s when your nose gets stuffy. And then it hardens inside your nose, and when you remove it, you start bleeding.

More than half of workers interviewed reported often relying on dry-cutting techniques both in the fabrication shops or during on-site installations in homes, and described a lack of wet saws, local exhaust ventilation, and wet cleaning procedures.

When we’re installing at someone’s house, and you need to cut something small and you don’t have access to water – that’s when you use your Makita [type of saw] to dry cut, and that’s when we use the vacuum. I mean, if someone tells you that they use water 100% of the time it is false.

Three-quarters (83%, 15/18) of workers noted that fabrication shops frequently smelled of acetone and resins used to glue engineered stone slabs together. Forty-four percent (8/18) of fabricators mentioned headaches and allergic reactions that they attributed to acetone and glue exposure, and one specifically mentioned the flammability of the mixed resin if left in a hot area.

Respondents often cited that employers transferred the cost of purchasing PPE onto workers with 50% (10/20) of workers providing their own PPE. One stated:

Not having a mask with filters. That’s a problem because we don’t have them at work. And we don’t get earplugs or safety goggles either. They [employers] don’t provide the equipment that we’re supposed to have. You have to keep buying them… And the boss doesn’t care. They say, ‘You have to take care of yourself, no one else will.’ And in the end, you’re like, ‘They’re right. Who’s looking out for you?’ But I think the employer should provide the protections required for work.

A majority of workers reported that their shop owners had installed machinery with wet-cutting capabilities to combat dust exposure (74%, 14/19), but some (36%, 5/14) stated this was only after an enforcement visit from OSHA regulators, rather than of their own volition. One worker stated, “That is a lot of money for the employer here, he [the employer] just put something like that [water cutting machine] because the city came, and I think they fined him.” As this quote underscores, workers cited the expense of installing water machines as a barrier for their employers. One worker reported that their employer had installed wet saws in the facility but failed to connect to a water source, leaving the machinery non-functional. It was perceived that employers used this method to appear as if they were following OSHA regulations to avoid fines.

#### 3.2.2 | Insufficient Training and Educational Resources

All workers were aware of at least some respiratory hazards related to cutting engineered stone, and specifically mentioned it could cause cancer, infections like tuberculosis, or breathing problems. However, none understood that it could cause “silicosis,” even when asked specifically about what problems breathing dust could cause, revealing a noticeable information gap. When asked if they had heard of regular preventative medical exams available to workers in their industry, less than half (45%, 9/20) had heard of chest radiographs as a commonly used medical exam to identify silicosis. One worker admitted that although he did not understand the disease process, he had a basic understanding that the material is dangerous, which he learned through informal means:

I don’t know much about it right now…but it is very dangerous. I have seen a few [silicosis] cases here from my friends, and I have also seen it on television, and the news, and I realized that it is dangerous. I do not know for sure what it is that enters or what is the dust that they see inside the lungs, I have to investigate it.

Similar to the above example, all workers we interviewed had gathered knowledge about dust-related lung disease through informal means. This included online videos (35%, 7/20), Spanish news segments (45%, 9/20), or word-of-mouth including stories of former coworkers or fabricators becoming ill (70%, 14/20). One worker shared his experience learning about lung disease associated with countertop fabrication:

I have heard about it, a boy died, a cutter, and they told him it was because of pneumonia. He arrived there [to the hospital] unable to breathe, with a cough, and they told him it was pneumonia, he never got out of there.

Personal experience knowing someone who has become ill was a motivating factor for workers to prioritize safety, as evidenced by one worker who stated, “We have protected ourselves more from the dust because we have seen friends who have gotten sick.”

In terms of targeted or formal education, six workers of our entire sample acknowledged the ramped-up outreach efforts of LACDPH and enforcement visits from Cal/OSHA as sources of information. These individuals demonstrated greater knowledge about workplace harms such as referencing the higher silica content of engineered stone as opposed to natural stone. However, most workers in our sample reported receiving no workplace education or training on how to work with engineered stone, often learning by doing (60%, 12/20). For instance, one worker stated the following, “We don’t know the materials and they don’t assign us a proper tool to work this… They never tell us things like, ‘This is a new material, you work it this way.’”

Workers were often not trained to understand the varying hazards of novel materials. Lack of training seemed to be somewhat dependent on the type of workplace, with well-established companies more likely to offer training compared to informal workshops. As one worker stated:

There may be classes, but the classes are given at the companies where you have good insurance to work. If you are working in a company that is in the dark, you are never going to have those classes.

The above quote confirmed that the worker felt a lack of access to training and proper protections. He implied that this is due to the limitations he, or immigrant workers, face in accessing “good” workplaces.

As for awareness of medical exams, workers reported being mostly unaware of legally required medical exams for employees in their industry (55%, 11/20). Nine workers confirmed hearing about chest radiographs as a medical exam for workers within the engineered stone fabrication industry, but they received this information from cautionary tales shared by friends and coworkers or the media. Only one of the nine workers reported receiving a chest radiograph in the last three years, but he did not clearly state if his medical providers were attempting to identify silicosis, although he did state that he had never been diagnosed with a respiratory illness.

#### 3.2.3 | Employment Vulnerability

Workers regularly felt powerless in improving workplace safety and agency to make change, mostly due to employer apathy towards promoting safe work practices (55%, 11/20), but also mentioned the high consumer demand for engineered stone and a lack of union representation. When workers advocated for increased safety practices for themselves and their coworkers, they were threatened with retaliation such as firing or deportation, or faced dismissal of their concerns including downplaying of the health risks associated with silica exposure. One worker shared that he feared advocating for increased safety protections because he witnessed a coworker get fired for doing so. He stated:

You feel afraid that the moment you choose to say something, they’re going to say, ‘There’s no more work for you.’…I heard a boy from Guatemala say, ‘They should give us masks, and this and that.’ A week went by, and I heard the secretary say, ‘These people talk a lot. We should fire them because they’re going to agitate the others’…He was fired.

Another worker similarly stated, “Many people are going through the same thing as me, who are depending on the boss and if you don’t like something, he fires you.” These quotes emphasize the fear of retaliation felt by many respondents.

Many workers described their employers as apathetic regarding safety, in that they would rather find replacement workers than improve safety. For instance, one worker stated:

Well, if I am being honest, I don’t think they [the employers] care. Do you understand? Because I’ve seen it. Just like with the people who have died or are sick, they don’t care about them anymore. They just find a replacement.

Another echoed the sentiment quoting his employer as saying, “You want to work? Work like this, if not, leave.” These statements highlight the vulnerability and powerlessness workers felt.

Even when employers installed wet-cutting machinery to mitigate silica dust exposure, they restricted its use. In the case of one worker, his previous employer limited the use of the wet saw to save money on their water bill, forcing workers to use water sparingly.

With the boss I had before, if you used too much water he would scold you, ‘Why did you use too much water? I’m going to get a more expensive bill. Try not to use too much water.’ He would limit us or check the way we worked with water in the manufacturing process.

Workers also felt powerless against the tastes of consumers to choose the materials they worked on. One worker noted that the widespread use of engineered stone left him feeling like he had no option but to work with the material.

That [engineered stone] has already entered the market… and we can’t do anything about it, that’s what they offer to people [consumers] and people ask for it, it’s what they see in the showrooms, it looks nice and that’s what they want.

Only one worker mentioned the lack of union representation as a barrier to protecting the stone fabricators in Los Angeles County.

We as marble workers or manufacturers do not even have a union, we have no one to represent us, so we have no choice but to work, even if there are risks, you must work.

This worker was unique in demonstrating awareness of the potential benefits of unionization, as other respondents focused on barriers to safety at an individual or workplace level.

#### 3.2.4 | Workforce Entrapment

Most respondents were recruited into stone countertop fabrication by family members already working in the industry (70%, 14/20), usually within a year or two of immigrating to the United States, evidenced by a median work tenure being only a few years shorter than the median years living in the United States. When asked about the barriers to leaving the industry given the health concerns, workers mentioned believing that their highly specific skillset was not transferable to another job (30%, 6/20). One stated, “It’s the only thing I know how to do here in this country…I’ve been working in this for 17 years and I don’t see myself doing any other job.”

Workers also felt that because they were obtaining a decent wage that had increased with experience, skill, and tenure in the industry, they felt apprehensive about the possibility of starting over in another industry where their presumed pay would be less (50%, 10/20). For instance, one stated:

Because of the pay if you leave the job, you are not going to earn the same in another job. Most people have been doing this for a long time, those who are changing jobs are those who are starting, those who are looking to improve themselves.

There was a perception that leaving the industry and starting out in a new one would have a direct negative impact on their financial situation.

The most cited factor prohibiting workers from leaving the industry was immigration status (55%, 11/20). Multiple workers expressed that they would leave the industry once they obtained legal status. One worker who was waiting to hear back on his visa application stated that if he had legal status he would “quit this job. I’d rather look for another job… even if it’s at McDonald’s, whatever, but not to be in the dust anymore because at my age being in the dust is not good anymore.” Others had similar views around legal status but coupled that with fears of mistreatment and low pay in other industries due to a lack of skills. For example, one worker stated:

You need a Social Security number, and we don’t have one…if I go to a new place, I’d be starting from zero, and you get paid very little… I’ve also asked them [other engineered stone fabricators] ‘Why don’t we change jobs?’ And they tell me, ‘I don’t want to start a new job. They mistreat you because you don’t know how to do the work. You’d have to start from zero and I have a family.’

Additionally, two workers (10%, 2/20) identified limited English proficiency as a barrier to changing industries. “If we don’t speak English—the language keeps us from doing many things here. So, it’s the only thing that’s available.”

However, workers did often transition among different stone fabrication workplaces to seek improved safety measures because of personal health issues such as a persistent cough, difficulty breathing, difficulty completing strenuous tasks or other health issues (60%, 12/20). One participant stated:

A month ago, I left the place where I was working because I started to have problems breathing. I tried to talk to the gentleman in the workshop about the same issue, that ventilation is needed, but he always says, ‘No, you can’t, I don’t have money.’ He never fixes anything, so that’s why I left. Where I am now… it is safer there because they have ventilation and a vacuum cleaner, that is why I moved there because it is safer.

Workers moved between shops seeking better working environments. Other workers were motivated to change fabrication shops after witnessing their employer’s negligent handling of a medical emergency (10%, 2/20). In one case, a worker attempted to get medical assistance for his coworker, but was forbidden by the employer who did not want attention brought to his shop’s lack of safety protocols.

I have a friend in that same place where I was, and that’s when I made the decision not to stay there;…, that man [friend] suddenly faints, as if he were dead,…I called the boss and said, ‘Look, this man got sick, I’m going to call the ambulance.’ And he said, ‘No, just pour water on his head. Don’t talk to the ambulance because they’re going to make a big problem for me here’…. if I depend on a person who is with me to talk to the ambulance, but the boss doesn’t give him permission, what kind of boss do we have?

These quotes emphasize that workers feel trapped in the countertop industry both by pull and push factors. However, many have transitioned laterally to workshops they perceived as having improved safety measures.

#### 3.2.5 | Worker-Identified Solutions

Workers had their own ideas about what changes they would like to see in their workplaces and the industry. The primary change fabricators wanted to see in their industry was widespread use of wet saws or “water systems” to limit dust accumulation in their shops (60%, 12/20). One stated, “The best thing to work this product is the water system because then you won’t breathe the dust.” When workers moved to shops owned by more responsible employers, they were more successful in advocating for the appropriate PPE and protections, especially when framing the suggestions as a cost-saving measure for the employer. One worker detailed how he successfully convinced his employer to purchase water-cutting machinery to avoid fines.

I started telling him [the employer], ‘Listen, we have to make these changes. You know that I don’t like the way we work here.’… The first thing is that I tell them [the employer] it’s for our safety, and secondly, I tell him, ‘It’s for your pocket.’ Because if the city or OSHA comes to visit, I tell him, ‘You’re going to get fined. They’re going to demand that you make all of these changes. And if we do them right now, there won’t be any issues.’…And he’s listened to me.

This was one of the few examples of a time when a worker did feel comfortable speaking up about safety concerns to their employer, and the response was positive.

Other worker-identified solutions included: employers reliably providing PPE (e.g., coveralls, respirators, ear protection, goggles, etc.) (30%, 6/20), updating laws to protect fabricators (15%, 3/20), and mandating adequate training to work with engineered stone (15%, 3/20). One worker acknowledged that employers are required to provide protections, “We’re supposed to get these things from the person we’re working with, but we have to buy them.”

Two workers who were also co-owners (and the only workers in their shop) mentioned that OSHA could be more helpful in clarifying to employers what they needed to do, instead of only providing pamphlets (10%, 2/20). The workers noted that as small-business owners they were happy to comply with OSHA regulations, but guidance was limited, and their financial resources were scarce compared to large fabrication shops.

Instead of them [Cal/OSHA] trying to just find irregularities, they should also offer some help, training, or something. In some way, I would like them to not be as strict with people like me who haven’t been in business as many years or who don’t have the resources bigger companies do to comply with these standards…I do want to comply with the requirements. That way, if I ever have people working for me, they will be okay. They will work well. But I would also like to receive a bit of support too.

This quote highlights that although this worker-turned-business-owner wanted to work responsibly, he felt a need for direction and support from the regulators.

Fifteen percent (3/20) of workers mentioned that increased enforcement, such as through tighter regulations, would be the motivating factors employers needed to make changes.

I think it has to be a law, mandatory, because if they don’t tell them, ‘You know what? If you don’t put this in, we’ll shut you down,’ then they put it in, but if they just tell them, ‘You need some fans,’ okay. It has to be a law that says they have to put that in, but if it’s not a law, they don’t care.

One worker went a step further, expressing his frustration, by suggesting a total ban of engineered stone across the United States as a response to the growing number of silicosis cases and other health issues related to working in fabrication shops.

I say, they should just stop producing these stones. In a first-world country like the United States, they shouldn’t allow these products to be sold. These governments are supposedly clean, unlike Mexico where there’s corruption. They’re supposed to stop the sale of this material here.

This quote exemplifies one of the few respondents that identified policy level solutions to prevent silicosis.

## 4 | Discussion

Among stone countertop workers in Los Angeles, our qualitative study revealed multiple personal, workplace-level, and societal influences on silica dust exposure that presented heightened risks for immigrant workers. Participants perceived the unique dangers of working with engineered stone products and expressed concerns about a lack of appropriate protections. While many workers recognized the potential health risks associated with silica dust, they often lacked specific knowledge about silicosis and effective prevention strategies, relying on informal sources of information. Challenges to safety included employers who did not take responsibility for worker safety, and language and immigration status barriers which prevented workers from effectively advocating for safer workplaces or moving to other industries.

Workers noted many examples of high dust levels in the workplace, a finding supported by prior studies that measured respirable crystalline silica dust concentrations in countertop fabrication worksites [26, 33, 34]. Workers noted visible white dust in the air, on their clothes and bodies, and inside their respirators as well as irritation of the eyes, throat, and nose as a result of consistent exposure to silica dust. These findings are consistent with previously published reports from investigations of countertop facilities in California and Texas that frequently found concentrations of respirable crystalline silica above the permissible exposure level and associated physical symptoms [19, 26, 33]. Workers also commonly noted smells of potentially toxic plastics, resins, and fillers. A potential explanation for this is the volatile organic compounds emitted when cutting engineered stone, identified by Ramkissoon et al. as styrene, with phthalic anhydride, benzene, ethylbenzene, and toluene [35].

Our results revealed that workers, generally, had limited knowledge of the risks of working with engineered stone and about employer requirements and worker protections under the Cal/OSHA Silica Standard [36], although most workers possessed a general awareness of its danger. Workers primarily obtained knowledge of the negative health effects of silica dust through informal means, suggesting that ongoing public health and workplace educational campaigns targeted to fabrication workers should be expanded. Additionally, public health campaigns that harness social media to improve health-related behaviors in Latino communities have been shown to be effective and should be utilized by public health agencies [37, 38]. However, this is in addition to the need for employer-sponsored on-the-job training about best work practices to avoid silica dust exposure.

Workers expressed the need to “take care of ourselves,” emphasizing the need to use their respirators and avoid dust, rather than arguing for change at the level of the workplace or society. This observation is similar to findings from a qualitative study of Latino immigrant workers in multiple industries that revealed workers often attempted to change their personal expectations of the work rather than identify ways to make the work itself safer [39].

Workers criticized employers for the unsafe working conditions present in fabrication shops. Workers stated that working conditions will not change until employers feel legally obligated to provide engineering controls and PPE, for instance. Workers believed that this lack of perceived responsibility for safety, in combination with a workforce that employers consider dispensable due to their precarious status in the US, de-incentivizes employers to improve safety conditions. This is despite the existence of OSHA Silica mandates requiring employers to provide training and adequate protections against high silica dust exposure [40]. However, we found that most workers received minimal to no on-the-job training and no education on the dangers of working with the material, or proper use of safety equipment such as respirator fit testing. This was consistent with prior data showing that only 20% of California countertop workers had ever been respirator fit tested [19]. Workers identified wet cutting machines which are effective in suppressing dust aerosolization [41–43], and cited the inconsistent provision of respirators which forced workers to provide their own. Although prior data on training patterns in this industry is minimal, prior research across industries suggests a lack of training for immigrant workers [44, 45], which partially explains the higher burden of occupational injury and illness among immigrant Latino workers [46].

At the societal level, our data are consistent with the idea that employers are more likely to neglect safety processes when employing undocumented immigrants due to a power differential where their employees would be less likely to complain about working conditions, for fear of retaliation. Immigrant workers in the United States, especially those who are undocumented, are particularly vulnerable and more likely to experience doing “dirty work” in precarious environments with no recourse for mistreatment or wage theft [47–49]. Our results re-iterate these findings, as some workers feared firing or further mistreatment for speaking out. Members of racial and ethnic minority groups are more likely to suffer workplace injustices and subsequent adverse psychological and physical health outcomes [50]. Precarious immigration status was one of the primary reasons workers continued in this industry despite knowing its danger, as likely their employers did not require legal US resident status.

Some policy changes have taken shape as a response to a growing number of silicosis cases among stone countertop workers at the state level in California [6], for instance, Cal/OSHA’s ETS and updated permanent standard [51, 52]. These standards strengthened the previous 2016 standard by no longer relying on 8-h time-weighted silica air sample monitoring and, instead, outright prohibiting all dry cutting [40]. In our sample, the majority, 68% (13/19), of workers engaged in dry-cutting, indicating that many workplaces would have to change to subsequently comply with the new standard, which was implemented just after we completed data collection. Future research is needed to assess employer compliance with these mandates.

In the global policy landscape, Australia has emerged as one of the most proactive nations in addressing silicosis among countertop workers. In response to increasing rates of silicosis in their country [5], Australia initiated a set of regulations including requiring health monitoring for stone fabrication workers, setting workplace exposure standards, and requiring the use of wet-cutting and dust extraction tools in the workplace [53]. However, despite these efforts and after scrutinizing exposure data, the Australia regulator recommended prohibition on the use of engineered stone in August 2023 [54], which was ultimately adopted in the country on July 2024 [55]. In addition, officials have made it a priority to address any cases that may come up in the future by establishing a network of support that promotes health monitoring for any workers who continue to work with materials may generate high levels of respirable crystalline silica. As one worker mentioned, a ban on the material would solve the problem in an upstream manner. However, without a national approach, a ban in one state like California, may push unsafe work across state lines. A handful of workers did suggest government action to develop stricter safety regulations and enforcement potentially including a ban on engineered stone slabs, as was done in Australia, as one respondent mentioned [56].

In the United States and California, our findings demonstrate that solutions will require a multi-pronged effort involving workers, employers, public health officials, medical professionals, relevant industry, and the public. The public health messaging is nascent and has predominantly centered using in-person outreach. Messaging should be expanded to include social media, given that workers we spoke to often sought information from online sources (e.g., YouTube, news clips, social media). In addition to ongoing increased regulatory and enforcement efforts, programs should be undertaken to incentivize employers, manufacturers, and industry representatives to coordinate high-quality hands-on training in the minimization of silica dust, compliant with the Cal/OSHA updated Silica standard. This should include language-concordant training for workers, which might also include indigenous languages. Leveraging culturally relevant educational materials to systematically provide accurate information about silicosis and recommended safety procedures will be critical to improving worker health. Lastly, development of a government silicosis screening program, or at minimum, easily accessible low-cost silicosis screening options for employers may also improve compliance with the Cal/OSHA Silica standard, help workers receive necessary medical care, and inform public health policies.

### 4.1 | Study Limitations

The study limitations should be considered in interpreting the results of the study. First, we primarily captured employee rather than employer perspectives and therefore our results lack that viewpoint. Although our sample does include two workers who happened to be co-owners of their fabrication shop, their entry into the industry as owners was only months-old at the time they were interviewed. Additionally, although efforts were made to reach workers from a diverse set of working environments, on more than one occasion, we interviewed several workers per site. This occurred because workers within the same site often felt encouraged to participate after a coworker’s participation. Another limitation is that study recruitment occurred in tandem with silicosis outreach by community partners, which meant that workers may have received a flyer or information session about silicosis before the interview, resulting in more informed responses during the interview. Additionally, althought our sample was small, we determined that data saturation had been reached through concurrent data analysis conducted alongside the interview process, confirming our sample was adequately sized. No quantitative sampling data (e.g., air quality data within a fabrication shop) was conducted to verify worker claims of mostly or at all times working with engineered stone. The focus of this study was to gather workers’ perspectives while ensuring their confidentiality, given the precarious nature of their work and legal status. Lastly, as a research project that was centered on community-partnered research, working with a vulnerable population meant that we had to make certain assurances to not divulge fabrication shop names and addresses so workers and employers were not in fear of our reporting them to Cal/OSHA or that their responses would lead to job loss.

## 5 | Conclusions

Our worker sample reported overwhelmingly unsafe working conditions characterized by frequent dry cutting of engineered stone, lack of water suppression methods, and inadequate provisions of respirators and engineering controls by employers. The results of this initial study provide a compelling explanation of pattern of disease, but more research is needed to improve diagnostic capabilities, design effective prevention strategies, treatment protocols, and public health responses. Based on conclusions drawn from worker testimonials presented earlier in this paper, workers want to feel safe and protected in the fabrication shops through the provision of personal protective equipment, adequate safety and health information, and training that would ensure their wellbeing.

Policy around silica exposure has evolved since the end of this study, but the number of cases is only expected to increase in the coming years with few options for treatment, most notably at advanced stages. Incorporating the perspectives of workers in local and state policy and ensuring those measures are being promoted by fabrication shop owners in practice are key to the future health and wellbeing of this worker population.

## Supplementary Material

Supplementary Appendix I

Additional supporting information can be found online in the Supporting Information section.

Appendix_I_Brief_Demographic_Survey_and_Interview_Protocol.

## Figures and Tables

**TABLE 1 |:** Interview guide questions.

Topic area	Questions
General overview of work environment	Can you tell me about your job in stone countertop fabrication and what it entails? What led you to start working in this industry? Can you walk me through a usual day at work? How is the work organized, for example what different types of tasks are done and by who?
Working conditions related to silica dust	Tell me about your working conditions related to dust exposure? Please give me details of your physical surroundings at work? Describe any protections that you regularly use, for example type of mask or respirator, and who provides this for you?
Hazard awareness of engineered stone	What do you know about the dangers of working with engineered (quartz) stone compared to natural stone countertops like marble or granite? If you are aware of health problems associated with these products, how did you learn about them? What can you tell me about the awareness of lung disease caused by cutting these stones (known as silicosis) among your friends and coworkers? Can you tell me about an example at work where there was formal information (e.g., from OSHA or your employer) provided about the danger of lung disease caused by inhaling dust while cutting these stones?
Personal experience with silicosis and work-related illness/injury	Some people experience breathing problems such as silicosis or asthma from working with engineered stones, how has this affected you? What have you heard about regular preventive medical exams (like an xray) to evaluate your lungs because of working in this industry? When a worker has a work-related illness or injury how does your boss respond? How does this person receive medical attention (e.g., worker’s compensation)?
Exposure reduction strategies	What have you heard about the different ways there are to make stone cutting safer? Some of the ways to prevent inhalation of particles include wet cutting, ventilation, respirator masks and testing the air quality. In your case, on a daily basis what are/were the challenges to maintaining safe cutting practices? Over the years that you have worked in this industry what changes have you seen in strategies to reduce dust exposure? What, if anything, have you tried to modify in your daily life at work to improve your own safety?
Perceived influence in the workplace	If you have had concerns about your work safety, what ideas do you have about making the work safer? Can you tell me about concerns you may have in discussing this with your employer? How would you describe the knowledge and responsiveness of your employer to the safety concerns around cutting stone?
Ease of leaving the industry	Have you ever thought about changing jobs or know anyone who has quit this job because of the health risks? What do you think are the reasons people would not try to or be able to change jobs? What is the impact of immigration status in the decision to leave or not leave your work in stone cutting? What is the impact of immigration status in improving safety in the workplace?
Assessment	If you were to talk with a friend or family member who was considering starting work in the stone-cutting industry, what would you tell them?

**TABLE 2 |:** Worker Characteristics.

Demographic or occupational characteristic	Overall (*n* = 20)
Demographic Characteristics (20)	*N* (%)
Median age, years [IQR]	44 [35–49]
Male	20 (100)
Hispanic/Latino	20 (100)
Country of origin	
Mexico	16 (80)
Central America, other	2 (10)
United States	2 (10)
Years since immigration (if non-US born), median [IQR]	24 [18–24]
Years worked in stone countertop industry, median [IQR]	20 [13–22]
Years worked with engineered stone, median [IQR]	10 [10–14]
Formally employed	13 (65)
Independent contractor/informal	7 (35)
**Healthcare access**	
Health Insurance	
Medi-Cal Complete^a^	9 (45)
Medi-Cal Restricted/Emergency^b^	5 (25)
Uninsured	6 (30)
Has usual source of care	15 (75)
**Work Practices and Protections**	
Employer reliably provides respirator (*n* = 18)	9 (50)
Engage in dry fabricating (*n* = 19)	13 (68)
Received formal safety training	1 (5)
Employer responsive to safety concerns	7 (35)
**Silicosis Awareness**	
Heard of silicosis (dust-related lung disease)	18 (90)
Word of mouth (friend/coworker) alone	7 (38)
Word of mouth & media/social media/internet	6 (33)
Formal education (e.g., doctor/public health outreach/workplace training)	5 (28)
Knows someone with silicosis	16 (80)
Aware of recommended medical testing	9 (20)
**Work-related symptom**	10 (50)
Respiratory (10)	8 (80)
Injury (10)	2 (20)
**Has considered leaving industry due to safety concerns (19)**	17 (90)

a Medi-Cal is California’s Medicaid system.

b Medi-Cal Restricted/Emergency is the limited scope Medicaid available to undocumented immigrants aged 26–50 years old who were not within the eligible age limits for full-scope Medi-Cal (< 25 years or > 50 years) at the time of the study.
