# Supplementary material for: Silica Hazards in Engineered Stone Countertop Production: Worker Experiences and Challenges in Los Angeles
Source: Am J Ind Med. Author manuscript; Available in PMC 2026 Jul 29. (PMC13419352; doi:10.1002/ajim.70010)
Supplement: Supplementary Appendix I [file NIHMS2186159-supplement-Supplementary_Appendix_I.docx]

## Supplemental Appendix I.

Participant ID: __________

**Understanding Stone Fabrication Workers' Perspectives on Safe Workspaces and Silicosis Hazards; a qualitative study**

**Semi-Structured Interview Guide (English):**

Introduction:

My name is __________ (first and last name) and I am a student with the Occupational Health Internship Program affiliated with UCLA. There has recently been an increase in the number of young men who work in the stone fabrication industry being diagnosed with a lung disease due to inhalation of silica dust, also known as silicosis. However, the risk factors for developing the disease have not been well studied. We would particularly like to learn about your experience with artificial (or quartz) stones such as those manufactured by Cosentino, Cambria, and Ceasarstone for example. Understanding your experience and perspective will hopefully help create changes that lead to safer working environments. We will also provide participants information on safer working practices from the California Department of Public Health. This is a confidential focused conversation that should take about an hour. Please understand that there are no right or wrong answers to any of the questions.

Demographic Questionnaire

| **Demographics** | | | |
| --- | --- | --- | --- |
| 1. Gender | □ Male  □ Female | 2. Do you identify as Hispanic/Latino? | □ Yes  □ No |
| 3. What is your primary language? | □ Spanish  □ English  □ Other, please specify: | 4. What country were you born in? |  |
| 5. In what year did you first move to the US? |  | 6. How many years have you worked in stone fabrication? (provide range of years, eg. 2001-2023) |  |
| 7. What is your employment arrangement | □ Formal Employee of a company  □ Independent contractor  □Other (describe) _________ | 8. How are you compensated for your work? | □ Hourly  □ Per slab  □ Per day  □ Other |
| 8. Do you have health insurance? | □ Yes  □ No | 9. What type of health insurance do you have? | □ Restricted (emergency) Medi-Cal  □ Full Medi-Cal  □ Medicare  □ Private insurance  □ Other  □ Don’t know  □ Refused |
| 13. Is there a place that you usually go to when you are sick or need advice about your health? (eg. doctor or clinic) | □ Yes  □ No |  |  |

If it is alright with you we are planning on recording this interview for documentation purposes but it will remain confidential.

(if Consent, recorders switched on)

**State date and time, and participant study ID number.**

**Interview questions**

***Grand tour question assessing general work environment.***

Can you tell me about your job working with stone countertops, what do you do?

- Follow up: What led you to start working in this industry?
- Follow up: Can you walk me through a usual day at work?
- Follow up: How is/was the work organized, for example what different type of tasks are done and by who?

***Working conditions and exposure to silica dust:***

Tell me about your working conditions related to dust exposure?

- Follow up: Please give me details of your physical surroundings at work?
- Probe questions
  - What does it look like? Sound like? Smell like?
  - Do you work mainly enclosed indoors or outdoors?
- Follow up: Describe any protections that you regularly use, for example type of mask or respirator, and who provides this for you?

**Engineered Stone hazard exposure awareness:**

What do you know about the dangers of working with artificial (quartz) stone such as Caesarstone as compared to natural stone countertops like marble or granite?

- Follow up: If you are aware of health problems associated with these products, how did you learn about them?
- Follow up: What can you tell me about the awareness of lung disease caused by cutting these stones (known as silicosis) among your friends and co-workers?
- Follow up: Can you tell me about an example at work where there was formal information (for example from OSHA or your employer) provided about the danger of lung disease caused by inhaling dust while cutting these stones?

**Personal experience with symptoms and medical care:**

Some people experience breathing problems such as silicosis or asthma from working with artificial stones, how has this affected you?

- Follow up: What have you heard about regular preventive medical exams (like an xray) to evaluate your lungs because of working in this industry?
- Probe: Has a doctor or medical professional ever diagnosed you with silicosis or another respiratory disease? Follow up: When a co-worker becomes sick or injured in the workplace, how does your employer respond?
  - How does that person get medical care? (e.g. Worker’s compensation)

**Exposure reduction strategies:** What have you heard about the different ways there are to make stone cutting safer?

- Follow up: Some of the ways to prevent inhalation of particles include wet cutting, ventilation, respirator masks and testing the air quality. In your case, on a daily basis what are/were the challenges to maintaining safe cutting practices?
- Follow up: Over the years that you have worked in this industry what changes have you seen in strategies to reduce dust exposure?
- Follow up: What, if anything, have you tried to modify in your daily life at work to improve your own safety?

**Assessing power in the work place (p):** If you have had concerns about your work safety, what ideas do you have about making the work safer?

- Follow up: Can you tell me about concerns you may have in discussing this with your employer?
- Follow up: How would you describe the knowledge and responsiveness of your employer to the safety concerns around cutting stone?
  - Can you give me an example?

**Immigration status and social mobility:** Have you ever thought about changing jobs or know anyone who has quit this job because of the health risks?

- Follow up: What do you think are the reasons people would not try to or be able to change jobs?

The following two questions are about immigration. If you feel uncomfortable you do not need ot answer

- Follow up: What is the impact of immigration status in the decision to leave or not leave your work in stone cutting.
- Follow up: What is the impact of immigration status in improving safety in the workplace.

**Concluding questions:**

If you were to talk with a friend or family member who was considering starting work in the stone cutting industry, what would you tell them?

Is there anything else about your working experience you wish to talk more about?

Thank you very much for everything you have shared. I appreciate your time. I will turn the recording devices off now.

(Provide Resources from CDPH and CASS Surveillance Study Flier)
